# A classification system of intraocular lens dislocation sites under operating microscopy, and the surgical techniques and outcomes of exchange surgery

**DOI:** 10.1007/s00417-016-3273-6

**Published:** 2016-01-27

**Authors:** Ken Hayashi, Soichiro Ogawa, Shin-ichi Manabe, Akira Hirata, Koichi Yoshimura

**Affiliations:** Hayashi Eye Hospital, 4-23-35 Hakataekimae, Hakata-Ku, Fukuoka, 812-0011 Japan

**Keywords:** Cataract surgery, Intraocular lens dislocation, Dislocation sites, Predisposing factor, Intraocular lens exchange surgery

## Abstract

**Purpose:**

The aim of this study was to examine the recent status of intraocular lens (IOL) dislocation according to a classification system based on vertical dislocation position, as well as the surgical techniques and outcomes of IOL exchange surgery.

**Methods:**

The medical records of 230 eyes from 214 consecutive patients who experienced IOL dislocation and underwent exchange surgery between 2006 and 2014 were reviewed. Vertical dislocation sites observed preoperatively under operating microscopy were examined, along with the surgical techniques and outcomes of IOL exchange.

**Results:**

Dislocation sites included (1) the anterior chamber (12.2 %), (2) pseudophakodonesis (19.1 %), (3) the anterior vitreous cavity (47.4 %), (4) trap door-like dislocation (dangling in the peripheral vitreous cavity; 16.1 %), and (5) the retinal surface (5.2 %). The IOL retained in the anterior segment was moved onto the iris by pulling it up through the limbal side ports with an anterior vitrectomy (67.8 %), or by pushing it up from the pars plana with an anterior vitrectomy (26.5 %), while the IOL dropped on the retina was lifting it up from the retina after pars plana vitrectomy (5.7 %). Mean uncorrected and distance-corrected visual acuity significantly improved postoperatively (*p* < 0.0001). Major complications included a marked elevation in intraocular pressure (7.8 %), pupillary capture (6.5 %), and vitreous hemorrhage (2.6 %).

**Conclusions:**

Based on the classification system, approximately 95 % of dislocated IOLs were retained in the anterior segment, and these IOLs were exchanged using an anterior approach through limbal incisions with an anterior vitrectomy. Visual acuity improved significantly, and serious complications were uncommon, probably because the IOL exchange techniques were standardized and simplified without pars plana vitrectomy.

## Introduction

Late dislocation of an intraocular lens (IOL) is a serious complication after cataract surgery [1–4]. Many surgical techniques are applied to management of dislocated IOLs, including exchanging or repositioning of the IOL with suturing to the sclera or iris, which is performed with a pars plana vitrectomy or an anterior vitrectomy [5–15]. The preferred technique differs depending on the surgeon. In general, posterior segment surgeons prefer the posterior approach with repositioning of the IOL and pars plana vitrectomy [5–13], while anterior segment surgeons prefer the anterior approach with exchange of the IOL through limbal incisions and anterior vitrectomy [4, 14, 15]. Because most IOLs currently implanted are single-piece acrylic IOLs without rigid loops, these IOLs are not suitable for repositioning by suturing to the sclera or iris in eyes without adequate capsular support. Accordingly, IOL repositioning procedures have recently gradually decreased, and IOL exchange procedures have become the predominant technique.

Although previous studies reported various sites of the dislocated IOL based on the horizontal position determined on slit-lamp microscopy [4, 12, 14], no classification system has been established to date. Because the classification system of the dislocation sites must be associated with the planned surgical technique, the sites should be determined based on the vertical position observed under an operating microscope with the patient in a supine position. Additionally, while pars plana vitrectomy was performed in most previous cases [5–8, 13], anterior vitrectomy was recently shown to be adequate even for eyes with a posteriorly dislocated IOL to the vitreous cavity [4, 12]. Thus, the need for pars plana vitrectomy should be taken into consideration for developing a classification of dislocation sites determined under an operating microscope.

We recently developed a classification system of IOL dislocation sites based on the vertical position determined under an operating microscope with special reference to the necessity of pars plana vitrectomy. Utilizing this classification system, we have performed many exchange surgeries for dislocated IOLs since 2006. The purpose of the present study was to investigate the recent status of late dislocation of the IOL based on our classification system, and to examine modified techniques and outcomes of exchange surgery for dislocated IOLs.

## Patients and methods

### Patients

This study was a retrospective interventional case series. The medical records regarding 269 eyes of 240 patients who consecutively underwent IOL exchange surgery due to IOL dislocation at the Hayashi Eye Hospital between April 2006 and June 2013 were reviewed. Only eyes with a minimum follow-up of 6 months from the time of IOL exchange surgery and eyes with serious dislocation of the IOL, i.e., those that had to be managed by explantation and scleral fixation of the posterior-chamber IOL, were included in the study. Exclusion criteria were eyes that had undergone corneal transplantation, eyes with other ocular pathologies requiring combined surgery, including glaucoma, retinal detachment, or endophthalmitis, or eyes with slight dislocation treated by repositioning of the IOL without suturing to the sclera or iris. The surgeons verified that the dislocated IOL was either encased in the capsule or outside of the capsule both before and during surgery. Two hundreds and nine eyes of 201 patients who developed an in-the-bag dislocation and 21 eyes of 21 patients who developed an out-of-the-bag dislocation were included in the analysis.

### Classification system of IOL dislocation sites

**Fig. 1:**
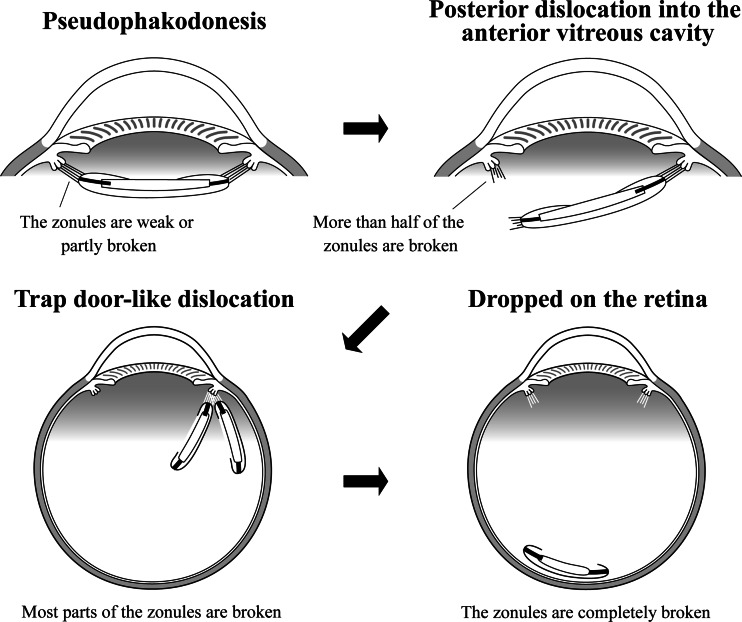
Schema of progression of in-the-bag dislocation of intraocular lenses (IOLs). In-the-bag dislocation of the IOLs generally progresses according to the number of the remaining zonules. When the zonules become weak or partly broken, pseudophakodonesis of the IOL begins. When probably more than half of the zonules are broken, the IOLs become tilted or sink into the anterior vitreous cavity. When most of the zonules are broken, the IOL dangles while connected with the remaining zonules in the peripheral vitreous cavity like a trap door. When the connection with the zonules or capsules breaks completely, the IOL drops onto the retina

**Fig. 2:**
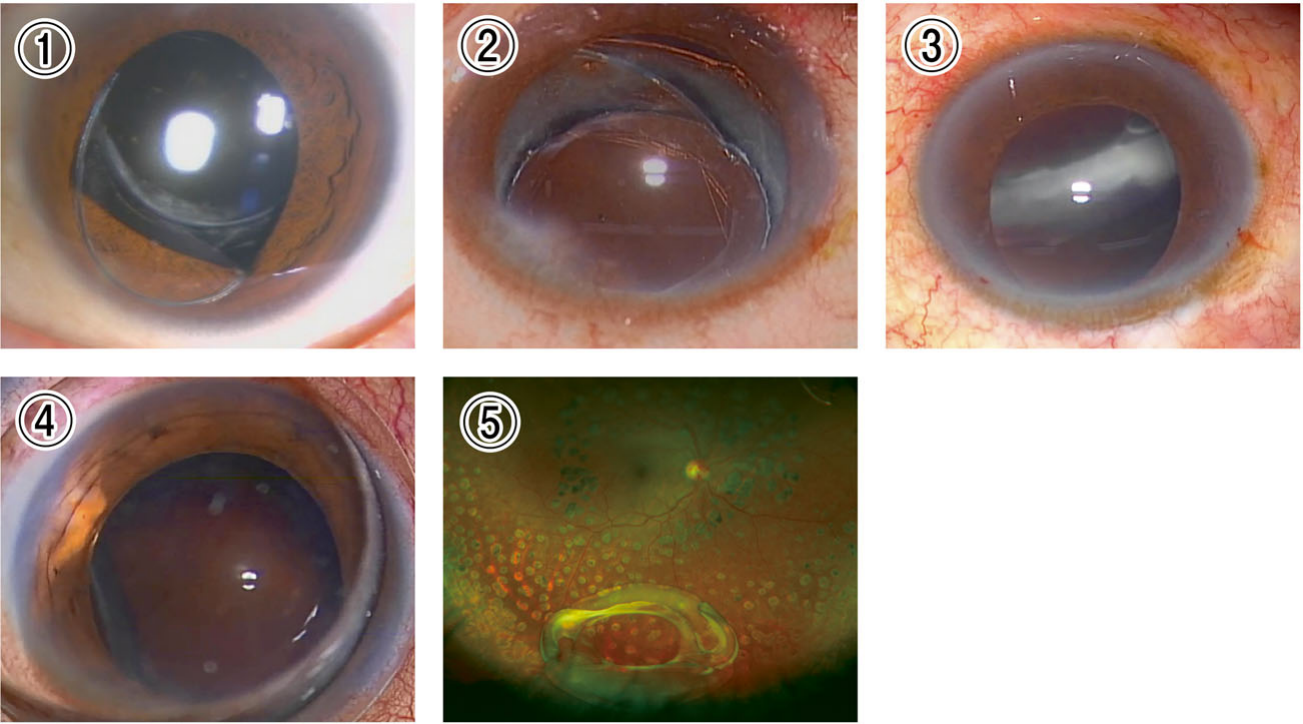
Classification system of the intraocular lens (IOL) dislocation sites. The dislocation sites are classified into five categories, which can be determined with the patient in a supine position under an operating microscope: (1) prolapse into the anterior chamber, (2) pseudophakodonesis, (3) posterior dislocation in the anterior vitreous cavity within the pupillary area, (4) trap-door-like dislocation (dangling in the peripheral vitreous cavity), and (5) dropped onto the retina

Dislocation of the IOL, typically in the in-the-bag dislocation, progresses vertically according to the degree of the remaining zonular or capsular support (Fig. 1). Weakening or breaking of a part of the zonules or capsules leads to pseudophakodonesis or tilt and sinking of the IOL, in which the IOL is observed within the pupillary area. When large portions of the zonules break, the IOL dangles in connection with the remaining zonules like a trap door in the peripheral vitreous cavity (trap-door-like dislocation). A perpendicularly dangling IOL is rarely seen within the pupillary area in a supine position, but when the upper zonules remain, the IOL is seen within the pupillary area on slit-lamp microscopy with the patient in a sitting position. When the connection with the zonules or capsules breaks completely, the IOL immediately drops onto the retina. According to this progression of dislocation, the dislocation sites were classified into the following five categories (Fig. 2): (1) prolapse into the anterior chamber, (2) pseudophakodonesis, (3) posterior dislocation in the anterior vitreous cavity within the pupillary area, (4) trap-door-like dislocation (dangling in the peripheral vitreous cavity, and (5) being dropped onto the retina. This classification system could be determined in patients in the supine position under an operating microscope.

### Surgical technique

Exchange surgeries of the dislocated IOLs were performed by four surgeons (KH, SM, AH, KY) using standardized surgical techniques. All IOLs were explanted and scleral fixation of new posterior-chamber IOLs was performed [4]. Before explantation, lifting up of the dislocated IOL onto the iris was performed using three techniques based on the classification of the dislocation site: (1) pulling up through two side ports at the limbus, (2) pushing up from the pars plana, or (3) lifting up from the retinal surface. For eyes showing pseudophakodonesis, prolapse into the anterior chamber, and slight posterior dislocation of the IOL into the vitreous cavity, a part (mostly haptics) of the IOLs were lifted up onto the iris using a bimanual hook or IOL forceps. For eyes with a trap-door-like dislocation and deep posterior dislocation into the vitreous cavity, after a trocar for 25-gauge vitrectomy was inserted in the pars plana where the zonules remained, the IOL was pushed up into the posterior chamber using a pick, and then lifted up onto the iris using a Sinsky hook or forceps through the limbus. For eyes in which the IOL had dropped onto the retina, 3-port pars plane vitrectomy was performed. The haptic of the IOLs was grasped and the IOL was lifted up to the posterior chamber using internal limiting membrane (ILM) peeling forceps or a pick, and then fixed onto the iris through the limbal side ports using a hook or IOL forceps. Thus, the former four sites of IOL dislocation were managed using an anterior approach, while only the dropped IOL on the retina was managed using a posterior approach.

After the dislocated IOL was removed using a forceps through an approximately 3.0- to 4.0-mm clear corneal incision or 6.0- to 7.0-mm scleral tunnel incision, scleral fixation of the posterior-chamber IOL was performed. After removing a minimal amount of the anterior vitreous using a vitreous cutter, most IOLs were sutured to the sclera using the ab externo method. In brief, 9–0 polypropylene looped sutures with a long curved or straight needle (Alcon Laboratories, Fort Worth, TX, USA) were hitched to the eyelets of the loop of the polymethylmethacrylate IOL or directly to the loop of the hydrophobic acrylic IOL. After a 26- or 27-gauge catheter needle was used to pierce the sclera into the ciliary sulcus approximately 1.5 mm posterior to the limbus, the end of the long needle was inserted into the cavity of the catheter needle, and then both needles were pulled out together from the eye. The IOL was then inserted into the posterior chamber, and bilateral sutures were drawn tight until the IOL was well-centered. Another superficial bite was taken in the sclera, and then one arm of the suture was cut and tied to the other arm of the suture; this was done on both sides. After instilling a miotic agent, the vitreous prolapse into the anterior chamber was removed using a vitreous cutter and vitreous strand was swept using a hook until the pupil became round. The IOL used for scleral suturing was a single-piece polymethylmethacrylate IOL (CZ70BD; Alcon), or a three-piece hydrophobic acrylic IOL (YA-65BB and VA70AD; HOYA, Tokyo, Japan). Some IOLs were fixed intrasclerally without sutures. After performing minimal anterior vitrectomy, both loops of the three-piece hydrophobic acrylic IOL were externalized from the sclerotomy sites using fine forceps or 24-gauge needles. The ends of the loops were buried in the half-thickness sclerotomy sites. The IOL used for intrascleral fixation without sutures was a 7.0-mm hydrophobic acrylic IOL with polyvinylidene difluoride loops (X-70 and NX-70; Santen, Osaka, Japan).

### Outcome measures

Data regarding in-the-bag and out-of-the-bag IOL dislocation and outcomes of the IOL exchange surgery were recorded. Data regarding IOL dislocation included predisposing conditions, dislocation site according to a classification system determined under an operating microscope, surgical techniques for IOL explantation, time interval between IOL implantation and exchange surgery, IOL type, and any complication during the cataract surgery.

Data regarding outcomes of the IOL exchange surgery included uncorrected and distance-corrected decimal visual acuity and refractive status preoperatively and at ∼ 6 months postoperatively, as well as postoperative complications. The refractive status was examined subjectively using an autorefractometer (KR-7100: Topcon, Tokyo, Japan). The manifest spherical equivalent value was determined as the spherical power plus half the cylindrical power. The endothelial cell density and central corneal thickness (CCT) were measured using specular microscopy preoperatively and at 6 months postoperatively, and the percentage of endothelial cell loss and increase in CCT was determined. Serious postoperative complications recorded included the occurrence of retinal detachment, a marked increase in intraocular pressure (IOP), corneal endothelial decompensation, cystoid macular edema, vitreous hemorrhage, pupillary capture, hypotony, choroidal detachment, chronic ocular inflammation, endophthalmitis, and redislocation of the IOL.

### Statistical analysis

Statistical analyses were performed to compare data between in-the-bag and out-of-the-bag IOL dislocation groups as well as between the preoperative and postoperative time points. Decimal visual acuity (VA) was converted to the logarithm of minimum angle of resolution (logMAR) scale for statistical analysis. The Mann–Whitney *U* test was used to compare the time interval between IOL implantation and exchange surgery between the groups, as well as VA, absolute manifest spherical equivalent value, corneal astigmatism, endothelial cell density, CCT between, before, and at 6 months after surgery, and other continuous variables. Dislocation site, surgical techniques used for IOL explantation, dislocated IOL type, and types of complications between the two groups, and other discrete variables were compared using Fisher’s exact test or the chi-square test for independent variables, where appropriate. Differences with a p value of less than 0.05 were considered statistically significant.

## Results

Of the 269 eyes that consecutively underwent IOL exchange surgery for dislocation, 39 eyes were excluded from analysis; 14 were eyes that underwent IOL exchange for reasons other than IOL dislocation, 12 were eyes that underwent combined surgery with other surgeries, including penetrating keratoplasty or glaucoma surgery, nine were eyes that underwent IOL exchange without scleral fixation, one eye that underwent corneal transplantation, one eye that underwent repositioning with suturing to the sclera, and two eyes that were lost to follow-up. Accordingly, 230 eyes of 214 patients were included in analysis.

Mean patient age (± standard deviation [± SD]) was 68.0 ± 12.9 years (range 28–92 years). There were 163 men and 67 women. Patients underwent examinations before and at ∼6 months after IOL exchange surgery. The patient characteristics of the in-the-bag and out-of-the-bag IOL dislocation groups are shown in Table 1. No statistically significant differences were found between the in-the-bag and out-of-the-bag dislocation groups with regard to age, sex, ratio of left to right eyes, time interval between cataract surgery and IOL exchange surgery, corneal astigmatism, or corrected VA. Mean arithmetic or mean absolute value of the manifest spherical equivalent and uncorrected VA preoperatively were significantly greater in the out-of-the-bag dislocation group than in the in-the-bag dislocation group (*p* ≤ 0.0012). Of the 230 eyes with serious dislocation of the IOL, 209 eyes (90.9 %) presented with in-the-bag dislocation, and 21 (9.1 %) presented with out-of-the-bag dislocation.

**Table 1:** Patient characteristics of the in-the-bag and out-of-the-bag intraocular lens (IOL) dislocation groups

Characteristics	In-the-bag dislocation	Out-of-the-bag dislocation	*p* value
	Group (*n* = 209)	Group (*n* = 21)	
Age (years)	70.4 ± 13.6	68.4 ± 12.6	0.4656
Sex (M/F)	149/60	14/7	0.6566
Left/Right	98/111	9/12	0.7239
Time interval (months)	128.9 ± 93.5	144.8 ± 138.4	0.9170
MRSE (D)			
Preoperatively	0.11 ± 5.10	4.78 ± 5.78	<0.0001*
Postoperatively	−2.22 ± 2.14	−1.66 ± 1.32	0.1389
Absolute MRSE (D)			
Preoperatively	3.28 ± 3.90	6.23 ± 4.07	0.0012*
Postoperatively	2.40 ± 1.92	1.68 ± 1.29	0.1106
Keratometric astigmatism (D)			
Preoperatively	1.29 ± 1.14	1.55 ± 1.21	0.3050
Postoperatively	1.64 ± 1.02	2.19 ± 2.39	0.5776
Uncorrected logMAR VA			
Preoperatively	1.09 ± 0.60	1.55 ± 0.58	0.0011*
Postoperatively	0.86 ± 0.51	0.82 ± 0.45	0.9311
Corrected logMAR VA			
Preoperatively	0.55 ± 0.59	0.54 ± 0.60	0.9904
Postoperatively	0.34 ± 0.52	0.45 ± 0.47	0.1491

*IOL* intraocular lens, *M* male, *F* female, *D* diopter, *MRSE* manifest spherical equivalent value, *logMAR VA* logarithm of minimum angle of resolution visual acuity

*Statistically significant difference

### Predisposing conditions for IOL dislocation

Of the 230 eyes with IOL dislocation (Table 2), the major possible predisposing conditions were pseudoexfoliation syndrome in 91 eyes (39.6 %), habitual eye rubbing or tapping in 47 (20.4 %), long axial length in 26 (11.3 %), and postvitrectomy in 24 (10.4 %). Of the 209 eyes with in-the-bag dislocation, major predisposing conditions were pseudoexfoliation syndrome in 90 eyes (43.1 %), habitual eye rubbing or tapping in 42 (20.0 %), long axial length in 24 (11.5 %), and postvitrectomy in 22 (10.5 %). Of the 21 eyes with out-of-the-bag dislocation, predisposing conditions were intraoperative capsular complications, including posterior capsule rupture or broken zonules, in five eyes (23.8 %), habitual eye rubbing or tapping in five (23.8 %), and secondary implantation of the IOL in three (14.3 %). The possible predisposing conditions differed significantly between the in-the-bag and out-of-the-bag dislocation groups (*p* < 0.0001).

**Table 2:** Number (%) of possible predisposing conditions for intraocular lens (IOL) dislocation

Conditions	In-the-bag dislocation	Out-of-the-bag dislocation	Overall
	Group (*n* = 209)	Group (*n* = 21)	(*n* = 230)
Pseudoexfoliation	90 (43.1 %)	1 (4.8 %)	91 (39.6 %)
Eye rubbing or tapping*	30 (14.4 %)	4 (19.0 %)	34 (14.8 %)
Atopic dermatitis	16 (7.7 %)	2 (9.5 %)	18 (7.8 %)
Long axial length	24 (11.5 %)	2 (9.5 %)	26 (11.3 %)
Postvitrectomy	22 (10.5 %)	2 (9.5 %)	24 (10.4 %)
Postglaucoma surgery	6 (2.9 %)	0 (0.0 %)	6 (2.6 %)
Postkeratoplasty	2 (1.0 %)	0 (0.0 %)	2 (0.9 %)
Operative complications†	16 (7.7 %)	5 (23.8 %)	21 (9.1 %)
Secondary implantation‡	0 (0.0 %)	3 (14.3 %)	3 (1.3 %)
Retinitis pigmentosa	6 (2.9 %)	2 (9.5 %)	8 (3.5 %)
Primary angle closure	2 (1.0 %)	0 (0.0 %)	2 (0.9 %)
Chronic uveitis	2 (1.0 %)	0 (0.0 %)	2 (0.9 %)
Unidentifiable	33 (15.8 %)	3 (14.3 %)	36 (15.7 %)

*Habitual eye rubbing or tapping, †Capsular complications at the time of cataract surgery, ‡Secondary implantation of the IOL

### IOL dislocation sites

Of the overall 230 eyes (Table 3), the major dislocation sites were posterior dislocation in the anterior vitreous cavity in 109 eyes (47.4 %), pseudophakodonesis in 44 (19.1 %), and trap-door-like dislocation in 37 (16.1 %). Of the 209 eyes with in-the-bag dislocation, major dislocation sites were posterior dislocation in the anterior vitreous cavity in 104 eyes (49.8 %), pseudophakodonesis in 42 (20.1 %), and trap-door-like dislocation in 36 (17.2 %), but being dropped onto the retinal surface occurred in only two eyes (1.0 %). Of the 21 eyes with out-of-the-bag dislocation, major dislocation sites were being dropped onto the retina in 10 eyes (47.6 %), dislocation in the anterior vitreous cavity in five (23.8 %), and prolapse into the anterior chamber in three (14.3 %). The incidence of the dislocation sites differed significantly between the in-the-bag and out-of-the-bag dislocation groups (*p* < 0.0001).

**Table 3:** Comparison of dislocation sites of intraocular lenses (IOLs) between in-the-bag and out-of-the-bag dislocation groups

Dislocation sites	In-the-bag dislocation	Out-of-the-bag dislocation	Overall
	Group (*n* = 209)*	Group (*n* = 21)*	(*n* = 230)
1) Prolapse into the AC	25 (12.0 %)	3 (14.3 %)	28 (12.2 %)
2) Pseudophakodonesis	42 (20.1 %)	2 (9.5 %)	44 (19.1 %)
3) Posterior dislocation into the anterior vitreous cavity	104 (49.8 %)	5 (23.8 %)	109 (47.4 %)
4) Trap-door-like dislocation	36 (4.8 %)	1 (4.8 %)	37 (16.1 %)
5) Dropped onto the retina	2 (1.0 %)	10 (47.6 %)	12 (5.2 %)

*AC* anterior chamber

*Statistically significant difference between the in-the-bag and out-of-the-bag dislocation groups (*p* < .0001)

### Surgical techniques of IOL explantation

**Fig. 3:**
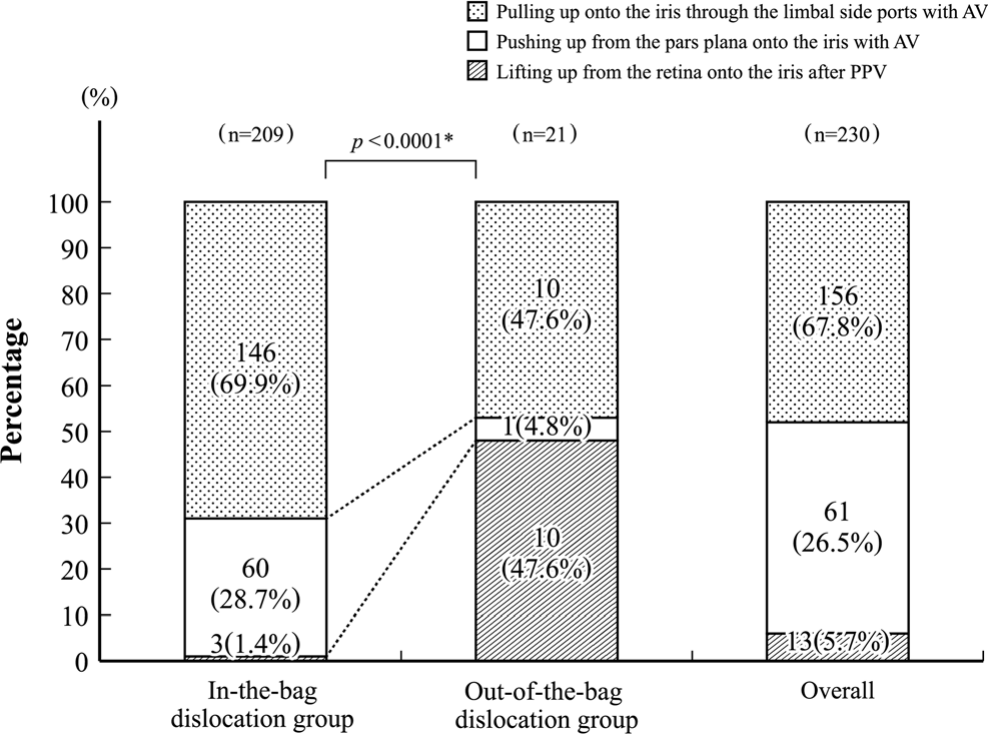
Explantation techniques of the dislocated intraocular lenses (IOLs) onto the iris. Explantation techniques of the dislocated IOL differed significantly between the in-the-bag and out-of-the-bag dislocation groups. *AV* anterior vitrectomy, *PPV* pars plana vitrectomy. *Statistically significant difference

Of the overall 230 eyes (Fig. 3), the IOL explantation techniques included pulling up onto the iris through limbal side ports in 156 eyes (67.8 %), pushing up from the pars plana with anterior vitrectomy in 61 (26.5 %), and lifting up from the retinal surface after pars plana vitrectomy in 13 (5.7 %). For eyes showing pseudophakodonesis (44 eyes, 19.1 %), prolapse into the anterior chamber (28 eyes, 12.2 %), and slight posterior dislocation of the IOL into the anterior vitreous cavity (84 eyes, 36.5 %), the IOLs were lifted up onto the iris using a bimanual hook or IOL forceps. For eyes showing a trap-door-like dislocation (37 eyes, 16.1 %) and deep posterior dislocation into the vitreous cavity (24 eyes, 10.9 %), the IOL was pushed up into the posterior chamber using a pick, and then lifted up onto the iris using a hook or forceps. For eyes in which the IOL had dropped onto the retinal surface (12 eyes, 5.2 %) and an eye in which the IOL could not be detected before pars plana vitrectomy (one eye, 0.4 %), the IOLs was lifted up from the retina after pars plana vitrectomy. The IOL explantation techniques were virtually consistent with the vertical IOL dislocation sites. The explantation techniques differed significantly between the in-the-bag and out-of-the-bag dislocation groups (*p* < 0.0001; Fig. 3).

### Postoperative complications and additional surgeries

Of the overall 230 eyes (Table 4), the major postoperative complications were a marked increase in IOP in 18 eyes (7.8 %), pupillary capture in 15 (6.5 %), vitreous hemorrhage in six (2.6 %), cystoid macular edema in four (1.7 %), and retinal detachment in three (1.4 %). Of the 209 eyes with in-the-bag dislocation, the major postoperative complications were a marked increase in IOP in 17 eyes (8.1 %), pupillary capture in 13 (6.2 %), and cystoid macular edema in four (1.9 %). Of the 21 eyes with out-of-the-bag dislocation, the major postoperative complications were vitreous hemorrhage in three eyes (14.3 %), pupillary capture in two (9.5 %), and a marked increase in IOP in one (4.8 %). The incidence of postoperative complications differed significantly between the in-the-bag and out-of-the-bag dislocation groups (*p* < 0.0001). Before IOL exchange surgery, two eyes had corneal decompensation with IOL dislocation, and three eyes had an epiretinal membrane.

**Table 4:** Postoperative complications in the in-the-bag and out-of-the-bag intraocular lens (IOL) dislocation groups

Complications	In-the-bag dislocation	Out-of-the-bag dislocation	Overall
	Group (*n* = 209)	Group (*n* = 21)	(*n* = 230)
Marked IOP increase	17 (8.1 %)	1 (4.8 %)	18 (7.8 %)
Pupillary capture	13 (6.2 %)	2 (9.5 %)	15 (6.5 %)
Vitreous hemorrhage	3 (1.4 %)	3 (14.3 %)	6 (2.6 %)
Hyphema	4 (1.9 %)	1 (4.8 %)	5 (2.2 %)
Retinal detachment	3 (1.4 %)	0 (0.0 %)	3 (1.3 %)
Cystoid macular edema	4 (1.9 %)	0 (0.0 %)	4 (1.7 %)
Transient corneal edema	5 (2.4 %)	0 (0.0 %)	5 (2.2 %)
Corneal epithelial defects	3 (1.4 %)	0 (0.0 %)	3 (1.3 %)
Bullous keratopathy*	2 (1.0 %)	0 (0.0 %)	2 (0.9 %)
Redislocation	1 (0.5 %)	0 (0.0 %)	1 (0.4 %)
Endophthalmitis	0 (0.0 %)	0 (0.0 %)	0 (0.0 %)

*IOP* intraocular pressure

*Bullous keratopathy present preoperatively

Of the overall 230 eyes (Table 5), major additional surgeries included vitrectomy for pathologies other than retinal detachment in eight eyes (3.5 %), IOL repositioning for pupillary capture in eight eyes (3.5 %), and glaucoma surgery in five (2.2 %). Of the 209 eyes with in-the-bag dislocation, major additional surgeries were IOL repositioning for pupillary capture in six eyes (2.9 %), glaucoma surgery in five (2.4 %), peribulbar injection of a long-acting steroid in five (2.4 %), and vitrectomy for retinal detachment in three (1.4 %). Of the 21 eyes with out-of-the-bag dislocation, the major additional surgeries were vitrectomy for epiretinal membrane or vitreous hemorrhage in four eyes (19.0 %), and IOL repositioning for pupillary capture in two (9.5 %). Descemet’s stripping automated endothelial keratoplasty was performed in two eyes with bullous keratopathy before IOL exchange, and pars plana vitrectomy was performed in three eyes with epiretinal membrane.

**Table 5:** Major additional surgeries of the in-the-bag and out-of-the-bag intraocular lens (IOL) dislocation groups

Surgery	In-the-bag dislocation	Out-of-the-bag dislocation	Overall
	Group (*n* = 209)	Group (*n* = 21)	(*n* = 230)
Vitrectomy for RD	3 (1.4 %)	0 (0.0 %)	3 (1.3 %)
Vitrectomy for pathologies other than RD*	4 (1.9 %)	4 (19.0 %)	8 (3.5 %)
Trabeculotomy	3 (1.4 %)	0 (0.0 %)	3 (1.3 %)
Trabeculectomy	2 (1.0 %)	0 (0.0 %)	2 (0.9 %)
DSEK	2 (1.0 %)	0 (0.0 %)	2 (0.9 %)
Repositioning of IOL for papillary capture	6 (2.9 %)	2 (9.5 %)	8 (3.5 %)
Peripheral iridectomy	4 (1.9 %)	0 (0.0 %)	4 (1.7 %)
Re-exchange of IOL	2 (1.0 %)	0 (0.0 %)	2 (0.9 %)
Anti-VEGF injection	2 (1.0 %)	0 (0.0 %)	2 (0.9 %)
Sub-Tenons’s injection of triamcinolone acetonide	5 (2.4 %)	1 (4.8 %)	6 (2.6 %)

*RD* retinal detachment, *DSEK* Descemet’s stripping endothelial keratoplasty, *VEGF* vascular endothelial growth factor

*Vitrectomy for vitreous hemorrhage and epiretinal membrane

### VA, refractive status, endothelial cell density, and central corneal thickness

**Fig. 4:**
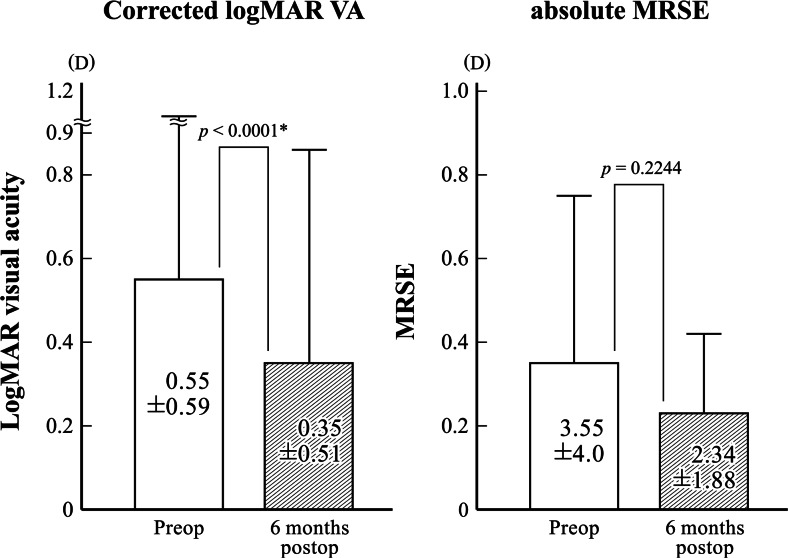
Changes in mean visual acuity and manifest spherical equivalent values. Mean uncorrected and distance-corrected visual acuity significantly improved postoperatively, while the mean absolute spherical equivalent value did not improve postoperatively. *Statistically significant difference

Mean corrected loMAR VA was 0.55 ± 0.59 preoperatively and 0.35 ± 0.51 at approximately 6 months postoperatively. Mean uncorrected and distance-corrected VA significantly improved postoperatively (*p* < 0.0001; Fig. 4). The mean absolute value of manifest spherical equivalent value was 3.55 ± 3.99 diopter (D) preoperatively and 2.34 ± 1.88 D at 6 months postoperatively. The mean arithmetic and absolute manifest spherical equivalent value (Fig. 4) did not improve postoperatively (*p* ≥ 0.2244). The endothelial cell density decreased significantly after IOL exchange (*p* < 0.0001), and the mean percentage of endothelial cell loss was 9.2 ± 22.5 %. The mean CCT did not change significantly at 6 months after IOL exchange surgery (*p* = 0.7056).

### Photograph of a representative eye that presented with trap-door-like dislocation

**Fig. 5:**
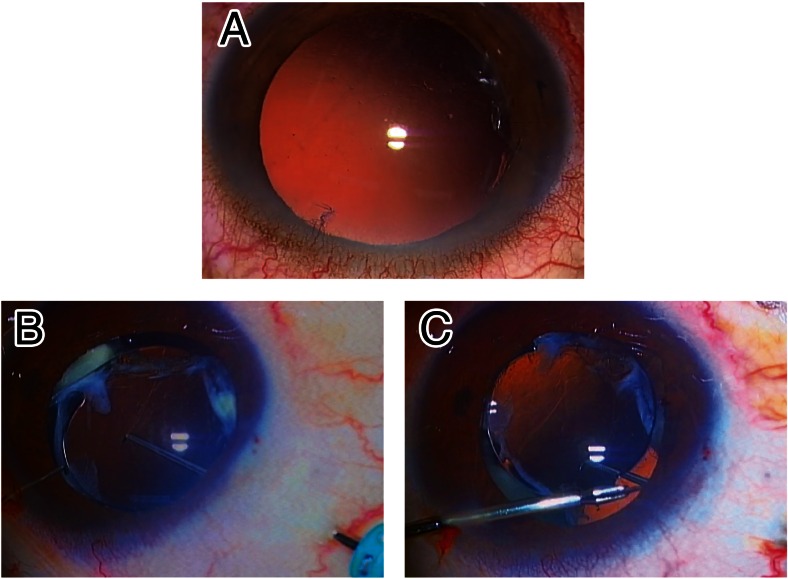
A representative eye that developed trap-door-like dislocation of intraocular lens (IOL). **a** Under an operating microscope, the IOL was difficult to see within the pupillary area because the IOL was dangling in the peripheral vitreous cavity while connected with the remaining zonules at the 10 o’clock meridian. After placing a trocar for 25-gauge vitrectomy into the pars plana at the 6 o’clock meridian, **b** the vertically dangling IOL was pushed up to the posterior chamber using a pick, and **c** was lifted onto the iris using an anterior capsulotomy forceps

A representative eye that developed the trap-door-like IOL dislocation is shown in Fig. 5. Under the operating microscope, the IOL was not observed within the pupillary area because the IOL was dangling in the peripheral vitreous cavity with the remaining zonules at the 10 o’clock meridian (Fig. 5a). After fixating a trocar for 25-gauge vitrectomy into the pars plana at the 10 o’clock meridian, the vertically dangling IOL was pushed up to the posterior chamber using a pick (Fig. 5b), and was lifted up onto the iris using an anterior capsulotomy forceps (Fig. 5c).

## Discussion

The findings of the present study revealed that recent states of IOL dislocation were different from those shown in our previous study [4]. The incidence of the in-the-bag IOL dislocation increased by approximately 91 %, while that of the out-of-the-bag dislocation decreased to 9 %. This is probably due to the fact the since the early 1990’s, almost all of the IOLs are implanted in the capsular bag, and most of the IOLs that developed IOL dislocation in the present study were implanted during this period. Furthermore, the major possible predisposing conditions for IOL dislocation also changed; the incidence of habitual eye rubbing or tapping associated with or without atopic dermatitis increased by 14.8 %. This is probably because the number of patients with ocular allergies has increased in our southern area of Japan, as well as throughout Japan [16].

The IOL dislocation sites were newly classified with regards to the degree of the remaining zonules or capsules. In this classification system, the vertical dislocation position of the IOLs was determined with the patient in a supine position under an operating microscope, and the horizontal position was not taken into consideration. According to this classification system, among the eyes with the in-the-bag dislocation, approximately 50 % of the eyes had the IOL dislocated in the anterior vitreous cavity, 5 % of eyes had a trap-door-like dislocation, and only 1 % of eyes had an IOL drop onto the retina. In contrast, among the eyes with the out-of-the-bag dislocation, 48 % of the eyes had an IOL drop onto the retina, and 24 % of eyes had the IOL dislocated in the anterior vitreous cavity. The differences in the dislocation sites suggests that the vertical position of the in-the-bag IOLs progresses depending on the number of the remaining zonules, and the IOLs drops onto the retina when the zonules are completely broken. Because the out-of-the-bag dislocated IOLs were maintained by the remaining capsule, the IOLs immediately drop onto the retina when separated from the capsule.

The vertical dislocation sites were associated with the IOL explantation technique. The IOLs with pseudophakodonesis, prolapse into the anterior chamber, and slight dislocation to the anterior vitreous cavity were pulled up onto the iris through limbal side ports, and the IOLs with deep dislocation in the vitreous cavity and trap-door-like dislocation were pushed up from the pars plana with anterior vitrectomy. Only the IOLs that dropped onto the retina were lifted up using an internal limiting membrane forceps or pick after pars plana vitrectomy. The IOLs in the former four dislocation sites were managed by an anterior approach, while the dropped IOLs onto the retina were managed by the posterior approach. In this series, approximately 95 % of the IOLs were explanted using an anterior approach, while only 5 % of IOLs that had been dropped onto the retina were explanted using a posterior approach after pars plana vitrectomy. Thus, we believe that eyes with IOL dislocation that need pars plana vitrectomy are uncommon, and, therefore, most eyes can be treated by anterior segment surgeons.

The incidence of postoperative complications was lower than that shown in our previous study, except for a marked increase in IOP and pupillary capture [4]. Because eyes that developed IOL dislocation also had a high incidence of pseudoexfoliation syndrome, a transient IOP elevation was unavoidable after IOL exchange surgery. In addition, pupillary capture frequently occurred after scleral suturing of the IOL, but it is thought that peripheral iridectomy is effective for preventing this complication. Indeed, after we began to perform peripheral iridectomy for most cases of scleral suturing of the IOL, the incidence of pupillary capture decreased. Furthermore, VA significantly improved, and the postoperative refractive error was slight. Thus, visual outcomes and complications after IOL exchange surgery were improved, probably because the surgical techniques were standardized based on the classification system of the vertical dislocation sites.

Many studies reported that IOL dislocation can be managed by exchange or repositioning of the IOL with or without suturing to the sclera or iris [5–15]. The surgical techniques vary depending upon the surgeon. Posterior segment surgeons prefer to reposition the preexisting IOL with suturing to the sclera or iris with pars plana vitrectomy [5–13], while anterior segment surgeons prefer to explant the IOL through the limbal incisions and fix a new IOL with anterior vitrectomy [14, 15]. Until recently, repositioning of the preexisting IOL with pars plana vitrectomy was the preferred technique because most cases with posterior dislocation were treated by posterior segment surgeons, and the type of the IOL was predominantly multipiece IOL with rigid loops. Because most IOLs currently implanted are single-piece acrylic IOLs, however, these IOLs are not suitable for repositioning with suturing to the sclera or iris in eyes without adequate capsular support. Furthermore, recent studies showed equivalent visual outcomes between the repositioning and exchange surgeries [17, 18]. For these reasons, we assume that exchange of the dislocated IOLs may become the predominant technique. Indeed, the present study showed that approximately 95 % of the dislocated IOL could be treated using the anterior approach with exchange of the IOL with infrequent postoperative complications.

In conclusion, we produced an IOL dislocation classification system based on the progression of vertical dislocation, which can be determined in a supine position under an operating microscope. The newly classified dislocation sites were associated with the IOL explantation technique. According to this classification system, approximately 95 % of dislocated IOLs could be exchanged using the anterior approach with explantation of the IOL, scleral fixation of a new IOL, and anterior vitrectomy. VA improved significantly after surgery, and postoperative complications were uncommon. Based on these results, we believe that most eyes with IOL dislocation can be treated safely without pars plane vitrectomy. Further study is required, however, to compare the visual outcomes and complications between anterior and posterior approaches within a randomized clinical trial.
